# Supplementary material for: VOLARE: visual analysis of disease-associated microbiome-immune system interplay
Source: BMC Bioinformatics. 2019 Aug 20;20:432. doi: 10.1186/s12859-019-3021-0 (PMC6701114; doi:10.1186/s12859-019-3021-0)
Supplement: Supplementary file 1 — Top table of microbe:cytokine relationships from case study 1. (PDF 39 kb) [file 12859_2019_3021_MOESM1_ESM.pdf]

Additional file 1: Table S1. Top table of microbe (Analyte1): cytokine (Analyte2) relationships from case study 1. F is from the partial F test of full model to reduced model, as described in Methods. P is the corresponding unadjusted p-value.

Rank	Analyte1	Analyte2	F	p
1	Mb_3	IL.22	52.2	1.19E-10
2	Mb_15	VEGF	26.6	1.88E-07
3	Mb_2	ICAM.1	21.7	1.27E-06
4	Mb_20	VEGF	21.2	1.55E-06
5	Mb_8	IL.15	19.0	4.04E-06
6	Mb_8	IL.1alpha	16.5	1.29E-05
7	Mb_15	IL.12.23p40	15.7	1.98E-05
8	Mb_2	IL.12.23p40	14.9	2.97E-05
9	Mb_8	IL.16	14.0	4.76E-05
10	Mb_7	IL.7	13.4	6.48E-05
11	Mb_6	IL.1alpha	11.7	1.65E-04
12	Mb_4	Calprotectin	10.7	2.97E-04
13	Mb_8	VEGF	10.6	3.11E-04
14	Mb_22	IL.22	10.2	3.85E-04
15	Mb_8	TNF.b	9.8	4.93E-04
16	Mb_8	GM.CSF	9.0	8.18E-04
17	Mb_22	sIgA	8.2	1.41E-03
18	Mb_21	IL.15	8.2	1.41E-03
19	Mb_15	IL.16	7.9	1.64E-03
20	Mb_20	IL.15	7.5	2.18E-03
21	Mb_10	IL.16	7.1	2.81E-03
22	Mb_2	TNF.b	7.1	2.97E-03
23	Mb_2	VCAM.1	7.0	3.10E-03
24	Mb_20	Calprotectin	6.9	3.25E-03
25	Mb_5	IL.22	6.9	3.36E-03
26	Mb_2	GM.CSF	6.8	3.64E-03
27	Mb_17	IL.1alpha	6.7	3.79E-03
28	Mb_14	IL.12.23p40	6.7	3.80E-03
29	Mb_19	sIgA	6.4	4.63E-03
30	Mb_11	VCAM.1	6.4	4.70E-03
31	Mb_14	VEGF	6.4	4.70E-03
32	Mb_15	IL.15	5.6	8.68E-03
33	Mb_2	IL.7	5.4	9.53E-03
34	Mb_21	VEGF	5.2	1.15E-02
35	Mb_14	ICAM.1	5.1	1.21E-02
36	Mb_16	sIgA	5.0	1.34E-02
37	Mb_8	IL.12.23p40	4.6	1.84E-02
38	Mb_15	ICAM.1	4.5	1.99E-02

39	Mb_4	CRP	4.3	2.18E-02
40	Mb_20	IL.12.23p40	4.3	2.29E-02
41	Mb_1	CRP	4.2	2.37E-02
42	Mb_2	IL.22	4.1	2.60E-02
43	Mb_12	CRP	4.0	2.81E-02
44	Mb_9	sCD14	3.8	3.38E-02
45	Mb_13	Calprotectin	3.7	3.60E-02
46	Mb_17	sIgA	3.7	3.66E-02
47	Mb_10	IL.1alpha	3.7	3.69E-02
48	Mb_4	VEGF	3.5	4.15E-02
49	Mb_13	IL.1alpha	3.5	4.24E-02
50	Mb_4	ICAM.1	3.5	4.28E-02
51	Mb_12	Calprotectin	3.5	4.30E-02
52	Mb_20	IL.16	3.5	4.38E-02
53	Mb_12	IL.1alpha	3.4	4.47E-02
54	Mb_8	Calprotectin	3.4	4.51E-02
55	Mb_4	IL.1beta	3.4	4.53E-02
56	Mb_7	IL.1alpha	3.4	4.80E-02
57	Mb_18	IL.16	3.4	4.81E-02
58	Mb_3	sIgA	3.3	4.86E-02
