# Supplementary material for: VOLARE: visual analysis of disease-associated microbiome-immune system interplay
Source: BMC Bioinformatics. 2019 Aug 20;20:432. doi: 10.1186/s12859-019-3021-0 (PMC6701114; doi:10.1186/s12859-019-3021-0)
Supplement: Supplementary file 2 — Structured interview outline (PDF 80 kb) [file 12859_2019_3021_MOESM2_ESM.pdf]

Additional file 2: Structured Interview Outline

Exploration of the potential value of multi-level visualization of linear model analysis in human systems biology

Background

- Introductions
- Linear model: Is there an association between a particular microbe and immune cell subset that differs by HIV status?
- Permutation testing allows us to identify statistically significant relationships, while controlling the false discovery rate
- The approach yields basic output: top N table, each with regression plot (*Show sample*)

1. **Scientific questions and data**
    a. How would you describe the data set?

    b. In an ideal world, what scientific questions does the data allow you to address?

    c. What challenges are there in analyzing the data to address the scientific questions?

    d. Which of the following describe your goals? (Munzner verbs here (p46), give interviewees a paper list: discover, present, enjoy, annotate, record (e.g. create video), derive; lookup, browse, explore, identify, compare, summarize). *Followup: why and how?*

2. **Current workflow and tools**
    a. What are the readily available/known tools for analyzing this data? How would you describe the workflow?

    b. Do you use any visualization tools? Are any interactive? Why or why not?

    c. What challenges are there in using readily available/known tools in analyzing the data?

    d. What are the pain points?

    e. What would you like to be able to do that you don't have tools to do?

    f. Are there specific bottlenecks to analyzing this data?

    g. What metrics would help you calibrate a particular result?

    h. What opportunities exist to improve your research based on improved analytical approaches? Which of these are most valuable?

3. **Communication**
    a. How would you like to share the results of analysis with your colleagues? In informal contexts? Formal contexts (presentations, publications)? With the general public? With scientists who are unfamiliar with one or both domains?

4. **Meta-questions**
    a. What about this research keeps you awake at night?

    b. Am I asking the right questions? Is there anything else I should be asking?

    c. Who else has similar data and needs?

    d. If you could …, what would it mean to your science?

    e. How would you characterize success in the analysis of this data?
